# Genetic Propensity for Delay Discounting and Educational Attainment in Adults Are Associated With Delay Discounting in Preadolescents: Findings From the Adolescent Brain Cognitive Development Study

**DOI:** 10.1111/gbb.70020

**Published:** 2025-03-27

**Authors:** Jill A. Rabinowitz, Nathaniel Thomas, Justin C. Strickland, John J. Meredith, I‐Tzu Hung, Renata B. Cupertino, Julia W. Felton, Brett Gelino, Bryant Stone, Brion S. Maher, Danielle Dick, Richard Yi, Victor Flores‐Ocampo, Luis M. García‐Marín, Miguel E. Rentería, Abraham A. Palmer, Sandra Sanchez‐Roige

**Affiliations:** ^1^ Department of Psychiatry Robert Wood Johnson Medical School, Rutgers University Piscataway New Jersey USA; ^2^ Department of Psychiatry and Behavioral Sciences Johns Hopkins University School of Medicine Baltimore Maryland USA; ^3^ Department of Psychiatry University of California San Diego La Jolla California USA; ^4^ Porto Alegre Rio Grande do Sul Brazil; ^5^ Center for Health Policy & Health Services Research Henry Ford Health Detroit Michigan USA; ^6^ Department of Mental Health Johns Hopkins Bloomberg School of Public Health Baltimore Maryland USA; ^7^ Department of Psychology University of Kentucky Lawrence Kansas USA; ^8^ Brain and Mental Health Program QIMR Berghofer Medical Research Institute Brisbane Australia; ^9^ School of Biomedical Sciences, Faculty of Health, Medicine and Behavioural Sciences The University of Queensland Brisbane Australia; ^10^ Institute for Genomics Medicine, University of California san Diego La Jolla California USA; ^11^ Department of Medicine Vanderbilt University Medical Center Nashville Tennessee USA

**Keywords:** ABCD, decision making, delay discounting, genetics, preadolescents, self‐regulation

## Abstract

Higher delay discounting (DD) (i.e., propensity to devalue larger, delayed rewards over immediate, smaller rewards) is a transdiagnostic marker underpinning multiple health behaviors. Although genetic influences account for some of the variability in DD among adults, less is known about the genetic contributors to DD among preadolescents. We examined whether polygenic scores (PGS) for DD, educational attainment, and behavioral traits (i.e., impulsivity, inhibition, and externalizing behavior) were associated with phenotypic DD among preadolescents. Participants included youth (*N* = 8982, 53% male) from the Adolescent Brain Cognitive Development Study who completed an Adjusting Delay Discounting Task at the 1‐year follow‐up and had valid genetic data. PGS for DD, educational attainment, impulsivity, inhibition, and externalizing behaviors were created based on the largest GWAS available. Separate linear mixed effects models were conducted in individuals most genetically similar to European (EUR; *n* = 4972), African (AFR; *n* = 1769), and Admixed American (AMR; *n* = 2241) reference panels. After adjusting for age, sex, income, and the top ten genetic ancestry principal components, greater PGS for DD and lower educational attainment (but not impulsivity, inhibition, or externalizing) were associated with higher rates of DD (i.e., preference for sooner, smaller rewards) in participants most genetically similar to EUR reference panels. Findings provide insight into the influence of genetic propensity for DD and educational attainment on the discounting tendencies of preadolescents, particularly those most genetically similar to European reference samples, thereby advancing our understanding of the etiology of choice behaviors in this population.

## Graphical abstract

Greater polygenic scores (PGS) for delay discounting and lower educational attainment were associated with greater delay discounting (i.e., preference for sooner, smaller rewards) among preadolescents most genetically similar to European ancestry reference panels.

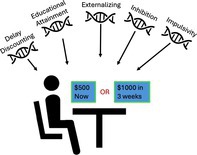

## 1 Introduction

Higher levels of delay discounting, defined as the preference for immediate, smaller rewards over larger, long‐term rewards, have been identified as a transdiagnostic risk factor [1, 2, 3, 4] for myriad negative outcomes such as substance misuse [5, 6, 7, 8, 9, 10, 11, 12], substance use disorder (SUD) severity [13, 14] and treatment outcomes [15], gambling [16], obesity [17], and lower educational attainment [18, 19]. Although delay discounting has previously been conceptualized as a stable behavioral trait, there is also evidence that delay discounting is contextually‐specific and a developmental phenomenon that rapidly increases from childhood to middle adolescence and stabilizes in late adolescence [20]. Given the numerous negative outcomes associated with increased sensitivity to delay, the identification of factors that predispose preadolescents to greater delay discounting is warranted. Such work may inform targeted preventative interventions earlier in development when delay discounting tendencies are still developing.

Twin studies have shown that genetics accounts for significant variability in phenotypic delay discounting [21, 22]. Indeed, genetic influences on delay discounting have been shown to account for 30% and 51% of the variance in phenotypic delay discounting at ages 12 and 14, respectively [23], with even larger heritability estimates observed in late adolescence (35%–66%) [24]. Within the last decade, there have been several genome‐wide association studies (GWAS) aimed at elucidating variants across the genome associated with complex traits (e.g., delay discounting) in adult populations [25, 26]. Although GWAS efforts focused on delay discounting have begun to uncover the genetic architecture of delay discounting in adult samples, there has been far less attention paid to understanding the genetic contributions to early adolescent delay discounting.

There are several reasons why genetic variants associated with delay discounting identified in adult populations may not predict delay discounting behaviors during preadolescence. First, it is possible that the genetic loci underpinning delay discounting in preadolescence, a developmental window characterized by significant brain maturation and plasticity, may be different than genes implicated in delay discounting in adult populations given the vast developmental changes that typify preadolescence [27]. Second, environmental exposures, such as scarcity and unpredictability, may influence the decision making behaviors of preadolescents in a different manner compared to adults. In support of this, previous work demonstrates that different environmental factors influence delay discounting at age 12 compared to age 14 [23], and unique environmental contributions to delay discounting are smaller at age 16 compared to age 18 [24]. In sum, differences in neurodevelopment and social environmental exposures may result in age‐related differences in the genetic contributions of delay discounting such that genetic variants associated with delay discounting in adult populations may not generalize to preadolescence. Identifying genetic contributors to delay discounting during this critical developmental stage may inform intervention or treatment targets that have downstream effects in reducing risk for maladaptive outcomes.

In addition to the lack of work that has examined genetic predictors of delay discounting in preadolescents, there is also a scarcity of work that has investigated whether the genetic underpinnings of delay discounting are shared with educational attainment. This construct may reflect cognitive and non‐cognitive processes, as well as behavioral states (e.g., externalizing behaviors, impulsivity) [28]. Among adults, there is evidence that delay discounting is strongly negatively correlated with IQ (*r*g = −0.63), years of education (*r*g = −0.67), and college attainment (*r*g = −0.93) [26]. Greater phenotypic delay discounting metrics have been linked to lower intelligence and grade scores [20, 29], poorer general cognitive abilities (e.g., executive function, working memory, poorer planning) [30, 31, 32, 33], and lower education levels in adolescent and adult samples [18, 19]. Using baseline data drawn from a nationally representative sample of preadolescents (i.e., Adolescent Brain Cognitive Development Study; ABCD), small, significant positive correlations were observed between delay discounting and impulsive traits such as negative urgency and positive urgency, as well as negative correlations with cognitive performance indicators [34]. Additional research has linked delay discounting to externalizing behaviors, although findings in this area have been mixed. For example, greater delay discounting has been associated with higher levels of externalizing symptoms among young adults [35, 36], and a greater odds of conduct disorder and oppositional defiant disorder among adolescents [37]. However, other research has observed no differences in delay discounting based on conduct disorder diagnostic status among preadolescents [38]. Discrepancies in findings across studies may be due to differences in developmental period (i.e., adolescence vs. adulthood), as well as variation in measurement approaches used to index delay discounting and cognitive and behavioral traits.

The goal of the current study was to determine whether genetic propensity for delay discounting, educational attainment, and behavioral traits (i.e., impulsivity, inhibition, and externalizing behavior) measured via polygenic scores (PGS) were associated with phenotypic delay discounting among preadolescents. Derived from GWAS, PGS are weighted, summed scores of genetic loci associated with a given phenotype and provide the opportunity to characterize genetic liability in traits measured in an independent, non‐overlapping sample [39]. Investigating whether polygenic liability for delay discounting and related traits is associated with phenotypic delay discounting in preadolescents has the potential to elucidate etiological pathways of delay discounting, as well as transdiagnostic processes that underpin other conditions.

## 2 Material and Methods

### 2.1 Participants and Demographic Information

The ABCD study is a landmark longitudinal study that aims to characterize changes in neural circuity, brain morphology, and behavioral measures that may be related to substance use engagement [40]. ABCD designated 21 data collection sites in which a stratified probability sampling of schools based on sex, socioeconomic status, race/ethnicity, and urbanicity were selected for each site to reduce systematic sampling biases in recruitment [41]. Participants' written assent and parental consent were obtained. Approval was obtained by the Rutgers Institutional Review Board to conduct the study.

Our analytic sample was limited to individuals who had valid genetic data and who completed the Adjusting Delay Discounting Task at the 1‐year follow‐up (i.e., when children were approximatly 10–11 years old) [42]. First, participants were removed from the ABCD dataset if they belonged to batch number 461 (*n* = 81), which was reported as problematic by the ABCD study. Next, participants who were missing delay discounting data were removed (*n* = 2354). Eight participants were in the same family, but attended different ABCD data collection sites and were excluded to facilitate modeling of the clustering of (1) participants within families, and (2) families within ABCD data collection sites. The resulting sample included 9433 participants, of whom 8982 were successfully matched to 1000 Genomes reference panels, as described below. The self‐reported racial/ethnic composition of the analytic sample included 6276 White, 1310 Black/African American, 746 Biracial, 141 Multiracial, 103 Asian, 40 Native American, and 3 Guamanian individuals. In terms of self‐reported ethnicity, 7145 indicated they were not Hispanic and 1726 reported that they were Hispanic.

Parents reported on their children's sex and race and selected an income category that best described their combined family income in the past year from the following options: 1 = less than $5000; 2 = $5000–$11,999; 3 = $12,000–$15,999; 4 = $16,000– $24,999; 5 = $25,000–$34,999; 6 = $35,000–$49,999; 7 = $50,000–$74,999; 8 = $75,000–$99,999; 9 = $100,000–$199,999; or 10 = greater than or equal to $200,000.

### 2.2 Phenotypic Delay Discounting Measurement

Delay Discounting was measured using an Adjusting Delay Discounting Task (ADDT) at the 1‐year follow‐up [42]. Youth participants selected between 2 hypothetical options: one that reflects receipt of a smaller, more immediate reward (e.g., $50) and one that reflects the receipt of a larger, future reward (e.g., $100). The ADDT is administered using seven randomly ordered blocks, including six choices in each, reflecting delays of 6 h, 1 day, 1 week, 1 month, 3 months, 1 year, and 5 years. The task automatically adjusts the choice options based on previous responses such that if the smaller, immediate reward is selected, the immediate reward decreases in value, whereas if the longer‐term, larger reward is selected, the immediate reward value increases.

In the current study, delay discounting was assessed using three approaches. First, we calculated *k*, or the degree of discounting based on the delay (D) [43]. Indifference points, or the points at which preferences for a smaller, more immediate reward are perceived as equal in value to a larger delayed reward, form the basis of a hyperbolic curve whereby a hyperbolic discounting function g(D) = 1/(1 + kD) is applied to calculate *k*. Given that *k* is often skewed, and to be consistent with the literature on delay discounting [44], we log transformed this variable. Second, we examined AUC, an atheoretical measure of area under the discounting curve. Third, given noted problems in traditional calculations of AUC, an alternative version of AUC calculation using log‐calculated delays was generated [45]. Higher AUC and log‐calculated AUC values indicate *lower* delay discounting (i.e., preference for larger, long‐term rewards), whereas higher values of *k* indicate greater or steeper delay discounting. Delay discounting tasks have been shown to have high strong internal and test–retest reliability and temporal stability among adolescents and adults [43, 46].

### 2.3 Genome‐Wide Association Studies

Delay discounting PGS was created based on a GWAS that included 134,935 individuals of European ancestry drawn from 23andMe [47] who completed the Monetary Choice Questionnaire (MCQ) [43]. Participants completed the online 27‐item MCQ as part of a larger survey to assess preference for smaller, immediate rewards versus larger, delayed rewards (see Supporting Information for questionnaire). The overall response pattern was used to derive temporal discounting functions (*k*), wherein higher values indicate a greater devaluation of delayed rewards (i.e., preference for immediate gratification). Participants with low response concordance or irregular response rates were excluded from the analysis (see Supporting Information). The SNP‐based heritability observed was 9.85% ± 0.57%.

PGS for impulsive personality traits were generated from a GWAS using 133,517 adults of 23andMe research participants of European ancestry based on self‐reports on the 20‐item Urgency‐Premeditation‐Perseverance‐Sensation Seeking‐Positive Urgency (UPPS‐P) scale [48, 49]. This instrument includes five subscales: *sensation seeking* or the propensity to pursue thrilling and novel stimuli; *negative urgency* or the tendency to engage in impulsive behaviors while in negative affective states; *positive urgency* or the predisposition to act impulsively while in a positive mood; *perseverance* or the extent to which one experiences challenges focusing on long, tedious tasks; and *premedita*tion or the ability to think about the repercussions of one's actions. We created PGS for each subscale of the UPPS‐P (i.e., sensation seeking, negative urgency, positive urgency, perseverance, and premeditation). Higher PGS reflect a greater genetic propensity for positive urgency, negative urgency, sensation seeking, and *lower* perseverance and premeditation.

We also created PGS for other impulsive traits using summary statistics from a GWAS [48] that was conducted based on self‐reports on the 30‐item Barratt Impulsiveness Scale—11 (BIS‐11) [50] from the same 23andMe participants referenced above. The BIS‐11 includes three subscales: *attentional impulsivity* or challenges with concentrating or focusing attention, *motor impulsivity* or difficulties with inhibiting physical actions, and *non‐planning impulsivity* or the tendency to act without thinking about the future. We created PGS for each subscale of the BIS‐11, with higher PGS reflecting a greater genetic propensity for attentional impulsivity, motor impulsivity, and non‐planning.

To create PGS for educational attainment, we leveraged the largest GWAS available on this phenotype, which included 3,037,499 individuals of European ancestry [51]. The largest GWAS on externalizing behaviors [52], which included approximately 1.5 million adults of European ancestry, was used to generate PGS for externalizing symptoms. This GWAS was conducted based on seven traits, including attention‐deficit hyperactivity disorder (ADHD), problematic alcohol use, lifetime cannabis use, lifetime smoking initiation, number of sexual partners, age at first sexual intercourse, and general risk tolerance. Higher PGS for externalizing behavior and educational attainment reflects greater genetic predisposition for externalizing symptoms and educational attainment, respectively.

### 2.4 Ancestry Assignment

We performed principal components analysis (PCA) on the unimputed ABCD genotype data (515,279 SNPs before quality control (QC)), which we merged with the 1000 Genomes [53] reference panel (18,026,714 SNPs before QC) to determine ABCD participants that were most genetically similar to European (EUR), African (AFR), and Admixed American (AMR) populations to facilitate genetic analyses by subgroup. First, we removed strand ambiguous SNPs, which have alleles that pair with each other in the DNA molecule (A/T, G/C), making it ambiguous whether the SNP was measured on the forward or reverse strand, from both datasets. This resulted in 480,164 SNPs in the ABCD sample and 15,510,861 SNPs in the reference panel. Next, we identified a set of 197,007 independent SNPs in the ABCD sample using PLINK [54, 55] by removing SNPs in regions of known LD (*high‐LD‐regions‐hg38‐GRCh38.txt* from the plinkQC R package) [56] and pruning for LD (‐‐indep‐pairwise 50 5 0.2). We extracted independent SNPs from the ABCD sample (*N* = 197,007) that overlapped with 1000 Genomes (*N* = 174,974). We then merged the two datasets after correcting chromosome, base pair position, and allele coding mismatches.

We used PC‐AiR [57] to create principal components (PCs) in the combined dataset while accounting for relatedness in the ABCD sample. The combined dataset was pruned again for LD using the *snpgdsLDpruning* function (method = “corr,” slide.max.bp = 10e6, and ld. threshold = sqrt(0.1)) from the SNPrelate R package [58] leaving 117,953 SNPs for PC calculation. We assigned participants to discrete genetic ancestry groups using Mahalanobis distance. Using the first 10 PCs, we calculated the Mahalanobis distance of each ABCD participant from each 1000 Genomes population and assigned participants to the population with the smallest Mahalanobis distance, consistent with approaches employed in previous studies [59]. In doing so, we observed 4972 who were genetically similar to EUR reference samples, 1769 who were genetically similar to AFR reference populations, and 2241 who were genetically similar to AMR reference panels.

### 2.5 Population Stratification Considerations

PCA was used to generate population stratification variables within genetic ancestry groups in PLINK 2.0 [54] using the Ricopili pipeline [60]. This procedure employs an orthogonal transformation to decrease the multi‐dimensional, genome‐wide SNP data into a smaller number of PCs. In alignment with previous recommendations [60], SNPs were considered for PCA if the minor allele frequency (MAF) was > 5%, Hardy–Weinberg equilibrium *p*‐value (HWE) was > 1.0e‐03, and missingness was < 2%. Strand ambiguous SNPs were removed. Additionally, SNPs in the major histocompatibility complex (6:25–35 Mb) or chromosome 8 inversion region (8:7–13 Mb). The dataset went through a round of LD pruning (indep‐pairwise 200,100 0.2) in PLINK to minimize redundancy with parameters recommended by Ricopili et al. [60] We identified ten within‐ancestry PCs that sufficiently captured population stratification in each of the genetic analysis groups.

### 2.6 Polygenic Score Creation

QC details of the imputed ABCD genotype data, which were used to create PGS, are documented in Fan et al. [61] PGS were created using PRS‐CS [62]. PRS‐CS uses a high‐dimensional Bayesian regression framework and applies a continuous shrinkage prior to SNP effect estimates. This approach infers posterior SNP weights for 1,120,697 common SNPs with a MAF ≥ 0.01 that are present across the HapMap3 [63] and 1000 Genomes Project Phase 3v5 [53] datasets. We used the 1000 Genomes Project Phase 3v5 reference panels, provided with the PRS‐CS software, to model linkage disequilibrium. Reference panels were matched to each genetic analysis group (i.e., the 1000 Genomes AFR reference panel was used for creation of the PRS in ABCD participants most genetically similar to AFR reference populations). PLINK version 2.0 [54] was used to calculate PGS, which involved summing all included SNPs weighted by the inferred posterior effect size for the effect allele. The PGS generated were subsequently standardized. The number of SNPs included in the resulting PRS ranged from 551,342 to 698,870.

### 2.7 Statistical Analyses

To determine the association of the PGS with phenotypic delay discounting outcomes (i.e., AUC, log‐calculated AUC, and log *k*), we conducted linear mixed effects models using the package *nlme* in R Version 4.3.1. Linear mixed effects analyses were conducted in each of the genetic ancestry groups (i.e., EUR, AFR, and AMR). Analyses adjusted for participant sex, age, household income, and the top ten within‐ancestry PCs. Across analyses, we used a nested random effects structure to account for (1) the clustering of participants within families and (2) the clustering of families within ABCD data collection sites.

**FIGURE 1:**
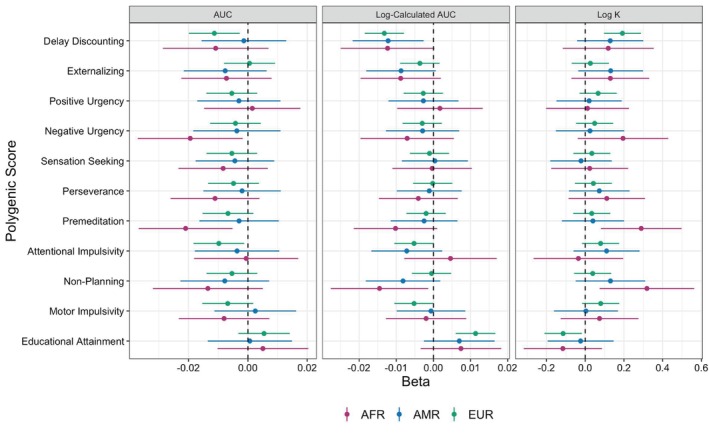
Associations of PGS with phenotypic delay discounting outcomes across genetic ancestry groups. Betas and 95% confidence intervals from linear mixed effects models predicting the three delay discounting outcomes are presented separately for each genetic ancestry group. Inferred statistical significance from 95% confidence intervals aligns with the raw *p*‐values, prior to FDR correction. AFR = Participants most genetically similar to African Reference Panel, AMR = Participants most genetically similar to Admixed American Reference Panel, EUR = Participants most genetically similar to European Reference Panel.

All regression coefficients reported from linear mixed effects models were standardized (M = 0, SD = 1). Standardization of the PGS facilitates interpretation of the PGS regression coefficients in units of standard deviations across genetically‐identified ancestral groups, provides an interpretable scale for the PGS, and avoids embedding implicit PGS mean comparisons across ancestry groups. Standardized betas and 95% CIs are presented in Figure 1. Marginal *r* ^2^ values were calculated using the *MuMIn* package in R to determine the amount of variance accounted for by the fixed effects when PGS were included in analytic models compared to when they were not. In interpreting the significance of the PGS, we adjusted for multiple testing by applying the Benjamini‐Hochberg [64] method within each genetic analysis group separately (11 predictors, 3 outcomes, 33 tests, false discovery rated‐adjusted *p*‐value ≤ 0.05).

**FIGURE 2:**
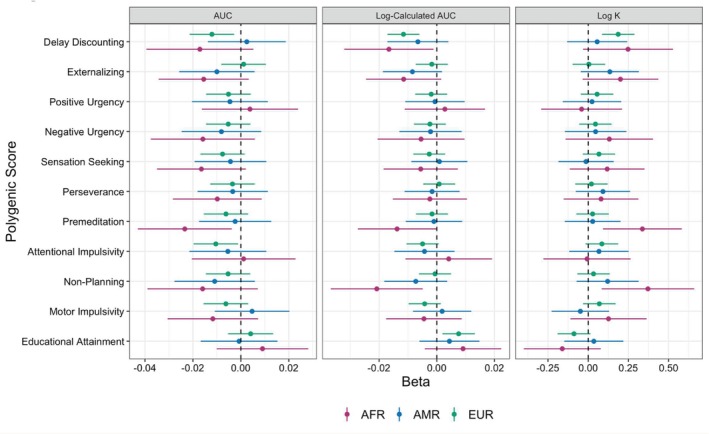
Associations of PGS with phenotypic delay discounting outcomes excluding more than one reversal across genetic ancestry groups. Betas and 95% confidence intervals from linear mixed effects models predicting the three delay discounting outcomes are presented separately for each genetic ancestry group. Inferred statistical significance from 95% confidence intervals aligns with the raw *p*‐values, prior to FDR correction. AFR = Participants most genetically similar to African Reference Panel, AMR = Participants most genetically similar to Admixed American Reference Panel, EUR = Participants most genetically similar to European Reference Panel.

**FIGURE 3:**
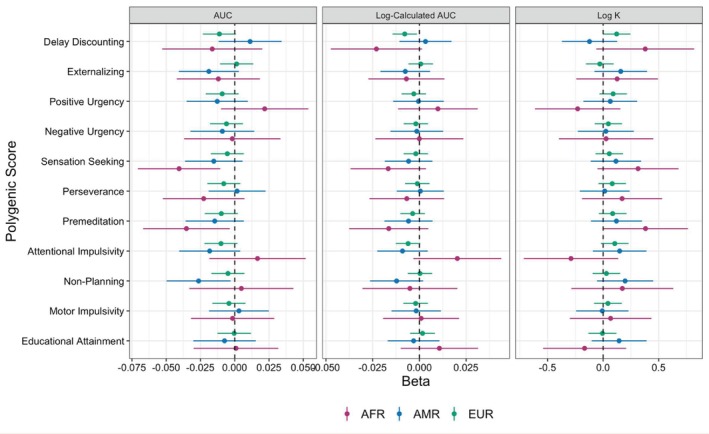
Associations of PRS with phenotypic delay discounting outcomes excluding any non‐systematic reversals across genetic ancestry groups. Betas and 95% confidence intervals from linear mixed effects models predicting the three delay discounting outcomes are presented separately for each genetic ancestry group. Inferred statistical significance from 95% confidence intervals aligns with the raw *p*‐values, prior to FDR correction. AFR = Participants most genetically similar to African Reference Panel, AMR = Participants most genetically similar to Admixed American Reference Panel EUR = Participants most genetically similar to European Reference Panel.

Data were evaluated for non‐systematic data rates consistent with best practice methods in delay discounting research [65]. Sensitivity analyses were applied to determine if results differed based on inclusion versus exclusion of non‐systematic delay discounting patterns. Filters were applied based on the bounce criteria as described by Johnson and Bickel [66]. The criteria state that data are flagged as non‐systematic if any indifference point (starting with the second delay) is greater than the preceding indifference point by a magnitude greater than 20% of the larger later reward (i.e., indicating that value increases by delay rather than decreases). Sensitivity analyses were conducted with a more conservative filter that removed data with any non‐systematic reversals and a more lenient criterion that removed data with more than one reversal. Standardized betas and 95% CIs from these analyses can be found in Figures 2 and 3.

## 3 Results

Participants included preadolescents (EUR *n* = 4972, *M* age = 9.94 years, SD = 0.63; AFR *n* = 1769, *M* age = 9.61 years, SD = 0.62; AMR *n* = 2241, *M* age = 9.91 years, SD = 0.63) of whom between 50.0% and 54.6% were male. Correlations of PGS and the phenotypic delay discounting outcomes are presented in Tables 1 and 2. Results from the primary analyses stratified by individuals that are most genetically similar to EUR, AFR, and AMR reference populations are presented below.

**TABLE 1:** Correlations of PGS in the EUR genetic ancestry group (*n* = 4972).

	*1*	*2*	*3*	*4*	*5*	*6*	*7*	*8*	*9*	*10*	*11*
1. Delay discounting	—										
2. Externalizing	**0.15** [0.13, 0.18]	—									
3. Positive urgency	**0.07** [0.04, 0.1]	**0.07** [0.04, 0.10]	—								
4. Negative urgency	**0.09** [0.06, 0.11]	**0.09** [0.06, 0.11]	**0.49** [0.46, 0.51]	—							
5. Sensation seeking	0.01 [−0.02, 0.04]	**0.08** [0.05, 0.11]	**0.15** [0.13, 0.18]	0.02 [−0.01, 0.05]	—						
6. Perseverance	**−0.05** [−0.08, −0.03]	−0.02 [−0.05, 0.00]	**0.08** [0.05, 0.11]	**0.08** [0.05, 0.11]	−0.01 [−0.04, 0.01]	—					
7. Premeditation	**0.05** [0.03, 0.08]	**0.06** [0.03, 0.09]	**0.28** [0.25, 0.31]	**0.24** [0.21, 0.26]	**0.08** [0.06, 0.11]	**0.30** [0.27, 0.32]	—				
8. Attentional impulsivity	**0.04** [0.02, 0.07]	**0.07** [0.04, 0.09]	**0.31** [0.28, 0.33]	**0.37** [0.34, 0.39]	**0.04** [0.01, 0.07]	**0.17** [0.14, 0.20]	**0.29** [0.26, 0.31]	—			
9. Non‐planning	**0.15** [0.12, 0.18]	**0.10** [0.07, 0.13]	**0.33** [0.31, 0.36]	**0.37** [0.35, 0.39]	0.03 [0.00, 0.05]	**0.22** [0.20, 0.25]	**0.45** [0.43, 0.47]	**0.42** [0.39, 0.44]	—		
10. Motor impulsivity	**0.06** [0.04, 0.09]	**0.14** [0.12, 0.17]	**0.29** [0.26, 0.31]	**0.27** [0.24, 0.30]	**0.16** [0.13, 0.19]	**0.13** [0.11, 0.16]	**0.32** [0.30, 0.35]	**0.29** [0.27, 0.32]	**0.39** [0.37, 0.41]	—	
11. Educational attainment	**−0.16** [−0.18, −0.13]	**−0.17** [−0.20, −0.14]	**−0.04** [−0.07, −0.02]	**−0.08** [−0.11, −0.05]	**0.03** [0.01, 0.06]	**0.03** [0.00, 0.06]	−0.02 [−0.05,0.01]	**−0.05** [−0.08, −0.03]	**−0.11** [−0.14, −0.09]	**−0.04** [−0.07, −0.01]	—

*Note:* Correlations involve the standardized PGS. Statistically significant correlations are marked in bold. 95% confidence intervals are reported in brackets.

**TABLE 2:** Correlations of PGS in the AFR (*n* = 1769) and AMR (*n* = 2241) genetic ancestry groups.

	1	2	3	4	5	6	7	8	9	10	11
1. Delay discounting	—	**0.26** [0.22, 0.30]	**0.26** [0.22, 0.30]	**0.29** [0.25, 0.33]	0.04 [0.00, 0.08]	0.04 [0.00, 0.08]	**0.20** [0.16, 0.24]	**0.23** [0.19, 0.27]	**0.32** [0.29, 0.36]	**0.22** [0.18, 0.26]	**−0.26** [−0.30, −0.22]
2. Externalizing	**0.14** [0.09, 0.19]	—	**0.21** [0.17, 0.25]	**0.24** [0.20, 0.28]	**0.20** [0.16, 0.24]	0.00 [−0.04, 0.04]	**0.13** [0.09, 0.17]	**0.18** [0.14, 0.22]	**0.25** [0.21, 0.28]	**0.22** [0.18, 0.26]	**−0.24** [−0.28, −0.20]
3. Positive urgency	**0.26** [0.21, 0.30]	**0.13** [0.09, 0.18]	—	**0.60** [0.57, 0.63]	**0.22** [0.18, 0.26]	**0.16** [0.12, 0.20]	**0.40** [0.36, 0.43]	**0.45** [0.42, 0.49]	**0.49** [0.45, 0.52]	**0.45** [0.41, 0.48]	**−0.17** [−0.21, −0.13]
4. Negative urgency	**0.35** [0.30, 0.39]	**0.16** [0.11, 0.20]	**0.61** [0.58, 0.64]	—	**0.11** [0.07, 0.15]	**0.17** [0.13, 0.21]	**0.35** [0.32, 0.39]	**0.50** [0.47, 0.53]	**0.51** [0.48, 0.54]	**0.41** [0.37, 0.44]	**−0.18** [−0.22, −0.14]
5. Sensation seeking	0.01 [−0.04, 0.06]	**0.15** [0.10, 0.19]	**0.17** [0.12, 0.21]	**0.07** [0.02, 0.12]	—	0.00 [−0.04, 0.04]	**0.13** [0.09, 0.17]	**0.13** [0.09, 0.17]	**0.09** [0.05, 0.13]	**0.21** [0.17, 0.25]	**−0.06** [−0.10, −0.02]
6. Perseverance	−0.05 [−0.09, 0.00]	−0.04 [−0.08, 0.01]	**0.09** [0.04, 0.13]	**0.09** [0.04, 0.13]	0.01 [−0.04, 0.06]	—	**0.34** [0.30, 0.37]	**0.22** [0.18, 0.26]	**0.29** [0.25, 0.32]	**0.19** [0.15, 0.23]	0.02 [−0.02, 0.06]
7. Premeditation	**0.24** [0.20, 0.29]	**0.14** [0.09, 0.19]	**0.42** [0.38, 0.45]	**0.40** [0.36, 0.44]	**0.12** [0.07, 0.16]	**0.34** [0.30, 0.38]	—	**0.36** [0.32, 0.39]	**0.51** [0.48, 0.54]	**0.41** [0.37, 0.44]	**−0.05** [−0.09, −0.01]
8. Attentional impulsivity	**0.30** [0.26, 0.35]	**0.10** [0.06, 0.15]	**0.47** [0.43, 0.51]	**0.55** [0.52, 0.59]	**0.09** [0.05, 0.14]	**0.18** [0.14, 0.23]	**0.40** [0.36, 0.44]	—	**0.56** [0.54, 0.59]	**0.42** [0.39, 0.46]	**−0.15** [−0.19, −0.11]
9. Non‐planning	**0.41** [0.38, 0.45]	**0.16** [0.11, 0.20]	**0.47** [0.44, 0.51]	**0.55** [0.52, 0.58]	**0.05** [0.01, 0.10]	**0.19** [0.15, 0.24]	**0.54** [0.51, 0.57]	**0.60** [0.57, 0.63]	—	**0.49** [0.46, 0.52]	**−0.19** [−0.23, −0.15]
10. Motor impulsivity	**0.17** [0.13, 0.22]	**0.17** [0.12, 0.21]	**0.40** [0.36, 0.44]	**0.33** [0.29, 0.37]	**0.18** [0.14, 0.23]	**0.16** [0.11, 0.21]	**0.40** [0.36, 0.44]	**0.36** [0.32, 0.40]	**0.44** [0.41, 0.48]	—	**−0.11** [−0.15, −0.07]
11. Educational attainment	**−0.21** [−0.25, −0.16]	**−0.17** [−0.22, −0.13]	**−0.14** [−0.18, −0.09]	**−0.17** [−0.22, −0.13]	0.01 [−0.04, 0.05]	**0.07** [0.02, 0.11]	**−0.11** [−0.16, −0.07]	**−0.12** [−0.17, −0.08]	**−0.20** [−0.25, −0.16]	**−0.10** [−0.14, −0.05]	—

*Note:* Correlations involve the standardized PGS. Statistically significant correlations are marked in bold. 95% confidence intervals are reported in brackets. Correlations in the AFR sample are presented left of the diagonal, whereas correlations in the AMR sample are presented to the right of the diagonal.

### 3.1 EUR Sample

After adjusting for age, sex, household income, and the top ten within‐ancestry PCs, the delay discounting PGS predicted log‐calculated AUC (*b* = −0.013, pFDR = 3.72E‐05, marginal *r* ^2^ = 0.50%) and log *k* (*b* = 0.192, pFDR = 9.08E‐05, marginal *r* ^2^ = 0.32%) such that greater delay discounting PGS was associated with greater delay discounting (i.e., greater sensitivity to immediate rewards) (Table 3). In contrast, greater PGS for educational attainment was linked to lower log‐calculated AUC (*b* = 0.011, pFDR = 0.001, marginal *r* ^2^ = 0.36%). See Figure 1 for standardized betas and 95% CIs.

**TABLE 3:** Associations of PGS with all phenotypic delay discounting data in the EUR genetic ancestry group (*n* = 4972).

PGS	AUC				Log‐calculated AUC				Log *K*			
	Beta	se	*p*	FDR‐adjusted *p*	Beta	se	*p*	FDR‐adjusted *p*	Beta	se	*p*	FDR‐adjusted *p*
Delay discounting	−0.011	0.004	**0.010**	0.082	−0.013	0.003	**1.13E‐06**	**3.72E‐05**	0.192	0.049	**9.08E‐05**	**0.001**
Externalizing	0.001	0.004	0.899	0.927	−0.004	0.003	0.179	0.400	0.027	0.049	0.588	0.669
Positive urgency	−0.005	0.004	0.215	0.400	−0.003	0.003	0.312	0.480	0.067	0.049	0.173	0.400
Negative urgency	−0.004	0.004	0.338	0.485	−0.003	0.003	0.264	0.442	0.049	0.049	0.320	0.480
Sensation seeking	−0.005	0.004	0.215	0.400	−0.001	0.003	0.694	0.764	0.034	0.049	0.483	0.580
Perseverance	−0.005	0.004	0.268	0.442	0.000	0.003	0.943	0.943	0.042	0.049	0.390	0.536
Premeditation	−0.007	0.004	0.124	0.342	−0.002	0.003	0.464	0.580	0.034	0.049	0.492	0.580
Attentional impulsivity	−0.010	0.004	**0.025**	0.139	−0.005	0.003	0.053	0.223	0.079	0.049	0.105	0.342
Non‐planning	−0.005	0.004	0.218	0.400	0.000	0.003	0.854	0.909	0.039	0.049	0.428	0.565
Motor impulsivity	−0.007	0.004	0.122	0.342	−0.005	0.003	0.054	0.223	0.080	0.049	0.103	0.342
Educational attainment	0.005	0.004	0.217	0.400	0.011	0.003	**3.31E‐05**	**0.001**	−0.114	0.049	**0.021**	0.137

*Note:* Analyses adjusted for participant age, sex, family income, and the top ten within‐ancestry PCs. Standardized beta coefficients are reported. Bolded values reflect statistically significant associations.

As noted above, we conducted sensitivity analyses using the bounce criteria as described by Johnson and Bickel [66]. Upon removing data with more than one reversal, 4418 youth (*M* age = 9.96 years, SD = 0.63, 53.6% males) who were genetically similar to EUR reference populations were retained. After removing observations with more than one reversal, higher PRS for delay discounting was linked to greater delay discounting outcomes (log‐calculated AUC *b* = −0.012, pFDR = 4.87E‐05; log *k b* = 0.186, pFDR = 5.18E‐03). Higher PRS for educational attainment was not linked to lower delay discounting after FDR correction (log‐calculated AUC *b* = 0.008, pFDR = 0.09) (Table 4). Upon exclusion of delay discounting data that had any non‐systematic reversals, a total of 2935 participants who were genetically similar to EUR reference populations were retained (*M* age = 9.98 years, SD = 0.63, 53.5% males). There were no significant main effects of any PGS on phenotypic delay after adjusting for multiple testing (Table 5). See Figures 2 and 3 for standardized betas and 95% CIs.

**TABLE 4:** Associations of PGS with phenotypic delay discounting excluding more than one reversal in the EUR genetic ancestry group (*n* = 4418).

PGS	AUC				Log‐calculated AUC				Log *K*			
	Beta	se	*p*	FDR‐adjusted p	Beta	se	*p*	FDR‐adjusted p	Beta	se	*p*	FDR‐adjusted *p*
Delay discounting	−0.012	0.005	**0.011**	0.090	−0.012	0.003	**4.87E‐05**	**1.61E‐03**	0.186	0.051	**3.14E‐04**	**5.18E‐03**
Externalizing	0.001	0.005	0.809	0.857	−0.002	0.003	0.544	0.681	0.004	0.052	0.942	0.942
Positive urgency	−0.005	0.005	0.274	0.519	−0.002	0.003	0.495	0.681	0.055	0.051	0.283	0.519
Negative urgency	−0.005	0.005	0.264	0.519	−0.002	0.003	0.398	0.597	0.045	0.051	0.381	0.597
Sensation seeking	−0.008	0.005	0.106	0.389	−0.003	0.003	0.355	0.597	0.067	0.051	0.194	0.457
Perseverance	−0.003	0.005	0.460	0.660	0.001	0.003	0.772	0.849	0.020	0.051	0.700	0.797
Premeditation	−0.006	0.005	0.189	0.457	−0.002	0.003	0.557	0.681	0.027	0.051	0.598	0.705
Attentional impulsivity	−0.010	0.005	**0.028**	0.182	−0.005	0.003	0.082	0.389	0.085	0.051	0.100	0.389
Non‐planning	−0.005	0.005	0.259	0.519	−0.001	0.003	0.831	0.857	0.033	0.051	0.520	0.681
Motor impulsivity	−0.006	0.005	0.188	0.457	−0.004	0.003	0.139	0.457	0.069	0.052	0.181	0.457
Educational attainment	0.004	0.005	0.394	0.597	0.008	0.003	**0.01**	0.090	−0.088	0.052	0.090	0.389

*Note:* Analyses adjusted for participant age, sex, family income, and the top ten within‐ancestry PCs. Standardized beta coefficients are reported. Bolded values reflect statistically significant associations.

**TABLE 5:** Associations of PRS with phenotypic delay discounting excluding any non‐systematic reversals in the EUR genetic ancestry group (*n* = 2935).

PGS	AUC				Log‐calculated AUC				Log *K*			
	Beta	se	*p*	FDR‐adjusted *p*	Beta	se	*p*	FDR‐adjusted p	Beta	se	*p*	FDR‐adjusted p
Delay discounting	−0.011	0.006	0.069	0.530	−0.008	0.003	**0.020**	0.530	0.122	0.063	0.056	0.530
Externalizing	0.001	0.006	0.822	0.911	0.001	0.003	0.828	0.911	−0.030	0.064	0.641	0.785
Positive urgency	−0.009	0.006	0.137	0.537	−0.003	0.003	0.371	0.736	0.090	0.063	0.156	0.537
Negative urgency	−0.006	0.006	0.324	0.736	−0.002	0.003	0.569	0.782	0.047	0.063	0.456	0.781
Sensation seeking	−0.005	0.006	0.378	0.736	−0.002	0.003	0.567	0.782	0.056	0.063	0.379	0.736
Perseverance	−0.008	0.006	0.190	0.537	−0.001	0.003	0.740	0.873	0.082	0.063	0.195	0.537
Premeditation	−0.010	0.006	0.113	0.530	−0.004	0.003	0.280	0.711	0.086	0.063	0.177	0.537
Attentional impulsivity	−0.010	0.006	0.104	0.530	−0.006	0.003	0.072	0.530	0.104	0.063	0.101	0.530
Non‐planning	−0.005	0.006	0.420	0.771	0.000	0.003	0.924	0.945	0.029	0.063	0.642	0.785
Motor impulsivity	−0.004	0.006	0.493	0.781	−0.002	0.003	0.547	0.782	0.043	0.064	0.497	0.781
Educational attainment	0.000	0.006	0.945	0.945	0.002	0.003	0.622	0.785	−0.006	0.064	0.922	0.945

*Note:* Analyses adjusted for participant age, sex, family income, and the top ten within‐ancestry PCs. Standardized beta coefficients are reported.

### 3.2 AFR Sample

Among individuals most genetically similar to AFR reference populations, there were no significant main effects of the PGS on the phenotypic delay discounting outcomes after adjusting for age, sex, family income, and the top 10 within‐ancestry PCs and accounting for multiple testing (Table 6). A total of 1326 participants were retained upon removing data with more than one reversal (*M* age = 9.94 years, SD = 0.62, 50.5% males), and 652 were retained upon removing data with any non‐systematic reversals (*M* age = 9.96 years, SD = 0.60, 49.4% males). Similar to results from the primary analysis, there were no significant main effects of any PGS on the phenotypic delay discounting outcomes after adjusting for multiple testing (Tables 7 and 8).

**TABLE 6:** Associations of PGS with all phenotypic delay discounting in the AFR genetic ancestry group (*n* = 1769).

PGS	AUC				Log‐calculated AUC				Log *K*			
	Beta	se	*p*	FDR‐adjusted p	Beta	se	*p*	FDR‐adjusted p	Beta	se	*p*	FDR‐adjusted *p*
Delay discounting	−0.011	0.009	0.234	0.513	−0.012	0.006	0.057	0.312	0.119	0.120	0.323	0.533
Externalizing	−0.007	0.008	0.354	0.556	−0.009	0.006	0.111	0.409	0.130	0.103	0.207	0.513
Positive urgency	0.001	0.008	0.857	0.943	0.002	0.006	0.765	0.901	0.012	0.109	0.914	0.951
Negative urgency	−0.019	0.009	**0.032**	0.214	−0.007	0.006	0.271	0.513	0.195	0.119	0.103	0.409
Sensation seeking	−0.008	0.008	0.280	0.513	0.000	0.005	0.951	0.951	0.023	0.101	0.820	0.933
Perseverance	−0.011	0.008	0.149	0.465	−0.004	0.005	0.454	0.651	0.111	0.101	0.271	0.513
Premeditation	−0.021	0.008	**0.010**	0.124	−0.010	0.006	0.076	0.361	0.289	0.106	**0.007**	0.124
Attentional impulsivity	−0.001	0.009	0.950	0.951	0.005	0.006	0.470	0.651	−0.035	0.118	0.765	0.901
Non‐planning	−0.013	0.009	0.155	0.465	−0.015	0.007	**0.031**	0.214	0.319	0.125	**0.011**	0.124
Motor impulsivity	−0.008	0.008	0.303	0.527	−0.002	0.006	0.722	0.901	0.074	0.103	0.473	0.651
Educational attainment	0.005	0.008	0.517	0.682	0.007	0.006	0.182	0.501	−0.116	0.103	0.262	0.513

*Note:* Analyses adjusted for participant age, sex, family income, and the top ten within‐ancestry PCs. Standardized beta coefficients are reported. Bolded values reflect statistically significant associations.

**TABLE 7:** Associations of PRS with phenotypic delay discounting excluding more than one reversal in the AFR genetic ancestry group (*n* = 1326).

PGS	AUC				Log‐Calculated AUC				Log *K*			
	Beta	se	*p*	FDR‐adjusted *p*	Beta	se	*p*	FDR‐adjusted *p*	Beta	se	*p*	FDR‐adjusted *p*
Delay discounting	−0.017	0.011	0.136	0.375	−0.017	0.008	**0.038**	0.250	0.247	0.143	0.086	0.320
Externalizing	−0.016	0.010	0.109	0.326	−0.011	0.007	0.087	0.320	0.200	0.120	0.098	0.324
Positive urgency	0.004	0.010	0.711	0.787	0.003	0.007	0.693	0.787	−0.041	0.128	0.750	0.798
Negative urgency	−0.016	0.011	0.156	0.390	−0.005	0.008	0.480	0.643	0.131	0.139	0.346	0.534
Sensation seeking	−0.016	0.009	0.085	0.320	−0.006	0.007	0.399	0.573	0.118	0.119	0.323	0.532
Perseverance	−0.010	0.009	0.301	0.522	−0.002	0.007	0.715	0.787	0.080	0.118	0.502	0.643
Premeditation	−0.023	0.010	0.021	0.174	−0.014	0.007	**0.050**	0.273	0.337	0.125	**0.008**	0.142
Attentional impulsivity	0.001	0.011	0.916	0.945	0.004	0.008	0.589	0.720	−0.007	0.138	0.958	0.958
Non‐planning	−0.016	0.012	0.177	0.390	−0.021	0.008	**0.012**	0.142	0.371	0.147	**0.013**	0.142
Motor impulsivity	−0.012	0.010	0.228	0.442	−0.004	0.007	0.507	0.643	0.126	0.121	0.299	0.522
Educational attainment	0.009	0.010	0.356	0.534	0.009	0.007	0.181	0.390	−0.161	0.122	0.189	0.390

*Note:* Analyses adjusted for participant age, sex, family income, and the top ten within‐ancestry PCs. Standardized beta coefficients are reported. Bolded values reflect statistically significant associations.

**TABLE 8:** Associations of PRS with phenotypic delay discounting excluding any non‐systematic reversal in the AFR genetic ancestry group (*n* = 652).

PGS	AUC				Log‐calculated AUC				Log *K*			
	Beta	se	*p*	FDR‐adjusted *p*	Beta	se	*p*	FDR‐adjusted *p*	Beta	se	*p*	FDR‐adjusted *p*
Delay discounting	−0.016	0.019	0.394	0.688	−0.023	0.012	0.086	0.523	0.379	0.225	0.114	0.523
Externalizing	−0.012	0.015	0.453	0.713	−0.007	0.010	0.514	0.713	0.126	0.187	0.512	0.713
Positive urgency	0.022	0.016	0.200	0.558	0.010	0.011	0.378	0.688	−0.229	0.196	0.262	0.665
Negative urgency	−0.002	0.018	0.923	0.983	0.000	0.012	0.999	0.999	0.027	0.217	0.904	0.983
Sensation seeking	−0.041	0.015	0.019	0.523	−0.017	0.010	0.128	0.523	0.314	0.186	0.114	0.523
Perseverance	−0.023	0.015	0.158	0.523	−0.007	0.010	0.519	0.713	0.170	0.184	0.370	0.688
Premeditation	−0.035	0.016	**0.046**	0.523	−0.016	0.011	0.152	0.523	0.382	0.195	0.070	0.523
Attentional impulsivity	0.017	0.018	0.370	0.688	0.020	0.012	0.113	0.523	−0.289	0.217	0.203	0.558
Non‐planning	0.005	0.019	0.806	0.983	−0.005	0.013	0.701	0.919	0.173	0.234	0.472	0.713
Motor impulsivity	−0.002	0.016	0.922	0.983	0.001	0.010	0.931	0.983	0.067	0.187	0.724	0.919
Educational attainment	0.001	0.016	0.954	0.983	0.011	0.011	0.327	0.688	−0.167	0.191	0.396	0.688

*Note:* Analyses adjusted for participant age, sex, family income, and the top ten within‐ancestry PCs. Standardized beta coefficients are reported. Bolded values reflect statistically significant associations.

### 3.3 AMR Sample

There were no significant main effects of the PGS for delay discounting, educational attainment, externalizing symptoms, inhibition, or impulsivity on phenotypic delay discounting after adjusting for age, sex, family income, and the top ten within‐ancestry PCs (Table 9). A total of 1903 participants were retained upon removing data with more than one reversal (*M* age = 9.93 years, SD = 0.64, 54.7% males), and a total of 1124 were retained after excluding observations with any non‐systematic reversals (*M* age = 9.95 years, SD = 0.63, 54.1% males). Similar to results from the primary analyses, there were no significant main effects of any PGS on phenotypic delay discounting after multiple testing adjustment (Tables 10 and 11).

**TABLE 9:** Associations of PGS with all phenotypic delay discounting in the AMR genetic ancestry group (*n* = 2241).

PGS	AUC				Log‐calculated AUC				Log *K*			
	Beta	se	*p*	FDR‐adjusted *p*	Beta	se	*p*	FDR‐adjusted *p*	Beta	se	*p*	FDR‐adjusted *p*
Delay discounting	−0.001	0.007	0.853	0.959	−0.012	0.005	**0.013**	0.433	0.129	0.087	0.141	0.647
Externalizing	−0.008	0.007	0.285	0.927	−0.009	0.005	0.069	0.647	0.131	0.085	0.126	0.647
Positive urgency	−0.003	0.007	0.673	0.959	−0.003	0.005	0.575	0.959	0.020	0.086	0.815	0.959
Negative urgency	−0.004	0.008	0.621	0.959	−0.003	0.005	0.564	0.959	0.025	0.090	0.785	0.959
Sensation seeking	−0.004	0.007	0.518	0.959	0.000	0.005	0.930	0.959	−0.022	0.081	0.787	0.959
Perseverance	−0.002	0.007	0.770	0.959	−0.001	0.004	0.803	0.959	0.073	0.080	0.365	0.959
Premeditation	−0.003	0.007	0.665	0.959	−0.003	0.005	0.584	0.959	0.040	0.082	0.628	0.959
Attentional impulsivity	−0.004	0.007	0.616	0.959	−0.007	0.005	0.141	0.647	0.110	0.087	0.206	0.757
Non‐planning	−0.008	0.008	0.309	0.927	−0.008	0.005	0.111	0.647	0.130	0.092	0.157	0.647
Motor impulsivity	0.002	0.007	0.723	0.959	−0.001	0.005	0.891	0.959	0.004	0.084	0.964	0.964
Educational attainment	0.001	0.007	0.928	0.959	0.007	0.005	0.152	0.647	−0.024	0.087	0.783	0.959

*Note:* Analyses adjusted for participant age, sex, family income, and the top ten within‐ancestry PCs. Standardized beta coefficients are reported.

**TABLE 10:** Associations of PRS with phenotypic delay discounting excluding more than one reversal in the AMR genetic ancestry group (*n* = 1903).

PGS	AUC				Log‐calculated AUC				Log *K*			
	Beta	se	*p*	FDR‐adjusted *p*	Beta	se	*p*	FDR‐adjusted *p*	Beta	se	*p*	FDR‐adjusted *p*
Delay discounting	0.002	0.008	0.765	0.928	−0.007	0.005	0.226	0.928	0.056	0.095	0.560	0.928
Externalizing	−0.010	0.008	0.214	0.928	−0.008	0.005	0.107	0.928	0.134	0.092	0.147	0.928
Positive urgency	−0.005	0.008	0.573	0.928	−0.001	0.005	0.905	0.928	0.024	0.093	0.800	0.928
Negative urgency	−0.008	0.008	0.340	0.928	−0.002	0.006	0.695	0.928	0.046	0.097	0.638	0.928
Sensation seeking	−0.004	0.008	0.571	0.928	0.001	0.005	0.855	0.928	−0.014	0.088	0.877	0.928
Perseverance	−0.003	0.007	0.651	0.928	−0.002	0.005	0.739	0.928	0.092	0.086	0.289	0.928
Premeditation	−0.002	0.008	0.757	0.928	−0.001	0.005	0.843	0.928	0.028	0.088	0.755	0.928
Attentional impulsivity	−0.005	0.008	0.509	0.928	−0.004	0.005	0.425	0.928	0.067	0.094	0.479	0.928
Non‐planning	−0.011	0.009	0.204	0.928	−0.007	0.006	0.193	0.928	0.121	0.098	0.222	0.928
Motor impulsivity	0.005	0.008	0.556	0.928	0.002	0.005	0.720	0.928	−0.049	0.091	0.594	0.928
Educational attainment	−0.001	0.008	0.928	0.928	0.004	0.005	0.410	0.928	0.035	0.094	0.710	0.928

*Note:* Analyses adjusted for participant age, sex, family income, and the top ten within‐ancestry PCs. Standardized beta coefficients are reported.

**TABLE 11:** Associations of PRS with phenotypic delay discounting excluding any non‐systematic reversals in the AMR genetic ancestry group (*n* = 1124).

PGS	AUC				Log‐calculated AUC				Log *K*			
	Beta	se	*p*	FDR‐adjusted *p*	Beta	se	*p*	FDR‐adjusted *p*	Beta	se	*p*	FDR‐adjusted *p*
Delay discounting	0.011	0.012	0.343	0.659	0.003	0.007	0.648	0.911	−0.123	0.127	0.338	0.659
Externalizing	−0.019	0.011	0.097	0.659	−0.008	0.007	0.270	0.659	0.158	0.121	0.196	0.659
Positive urgency	−0.013	0.011	0.267	0.659	0.000	0.007	0.943	0.949	0.064	0.123	0.604	0.905
Negative urgency	−0.009	0.012	0.450	0.742	−0.001	0.007	0.848	0.949	0.024	0.128	0.851	0.949
Sensation seeking	−0.015	0.011	0.159	0.659	−0.006	0.007	0.378	0.659	0.115	0.116	0.325	0.659
Perseverance	0.002	0.011	0.872	0.949	0.001	0.006	0.938	0.949	0.014	0.115	0.901	0.949
Premeditation	−0.015	0.011	0.183	0.659	−0.006	0.007	0.380	0.659	0.119	0.117	0.313	0.659
Attentional impulsivity	−0.018	0.011	0.113	0.659	−0.009	0.007	0.195	0.659	0.148	0.123	0.232	0.659
Non‐planning	−0.026	0.012	**0.032**	0.659	−0.012	0.007	0.098	0.659	0.197	0.129	0.133	0.659
Motor impulsivity	0.003	0.011	0.783	0.949	−0.002	0.007	0.800	0.949	−0.008	0.120	0.949	0.949
Educational attainment	−0.007	0.012	0.525	0.825	−0.003	0.007	0.663	0.911	0.144	0.126	0.259	0.659

*Note:* Analyses adjusted for participant age, sex, family income, and the top ten within‐ancestry PCs. Standardized beta coefficients are reported. Bolded values reflect statistically significant associations.

## 4 Discussion

Advances in molecular genetics and larger discovery samples have enabled the identification of genetic polymorphisms associated with variability in delay discounting and related traits [48, 52]. Most studies examining the genetic contributions of delay discounting and correlates to phenotypic delay discounting have examined these relations in adult samples [67]; however, it is unclear whether findings from these studies generalize to preadolescence when delay discounting is still developing [17]. Moreover, some researchers have suggested that preadolescents do not possess the cognitive maturity required to effectively complete monetary choice tasks, which may compromise the conclusions drawn [68]. However, the current findings support the validity of monetary choice behavioral tasks in preadolescents by showing that genetic influences of delay discounting and educational attainment identified in adult populations can be used to predict performance on these tasks earlier in life. These findings highlight that genetic loci underpinning this construct may be stable.

In analyses involving youth most genetically similar to EUR reference populations, greater polygenic propensity for greater delay discounting and lower educational attainment was associated with greater delay discounting (i.e., preference for sooner, smaller rewards), although the PGS accounted for a very small amount of variance in the outcomes (0.32%–0.50%). The observed effect sizes, however, are comparable to estimates from a recent study that examined the effect of delay discounting PGS on delay discounting outcomes in adults aged 24–28 (0.29%–0.80%) [67]. Our findings highlight that the polygenic architecture of delay discounting and educational attainment in adults can be reflected in performance on a behavioral task aimed at assessing delay discounting as early as preadolescence. Delay discounting paradigms often require participants to maintain active representations of the value of the rewards, as well as focus on goal‐relevant information. Many of the cognitive and behavioral processes linked to genetic propensity for educational attainment may govern approach tendencies and decision making that influence performance on decision making tasks [26, 69]. For example, deciding between the value of rewards often requires affect regulation (e.g., managing excitement regarding the potential receipt of an immediate reward), choice evaluation (e.g., determining the opportunity cost of not selecting the immediate reward), recall of previous decisions, and consideration of future choices. The numerous concurrent processes involved in completing this task, including maintaining and updating working memory and the recruitment of executive function resources, have all been linked to greater educational attainment [70]; these processes may partially explain the association between polygenic propensity for educational attainment with phenotypic delay discounting. Polygenic influences associated with educational attainment and delay discounting may also be associated with shared neural substrates that influence delay discounting tendencies. Indeed, there is evidence that regional gray matter volume in the left dorsolateral prefrontal cortex was associated with both delay discounting and academic performance [71], suggesting a common neural basis.

We did not find significant associations between the PRS for externalizing behavior and phenotypic delay discounting outcomes. The underlying structure of the externalizing common factor GWAS, which includes mostly substance use phenotypes measured in adult samples, may explain the observed null associations. There is evidence that genetic variance in substance use phenotypes varies across developmental periods, and environmental factors are generally more influential during earlier developmental periods [72]. Thus, genetic influences on adult substance use behaviors, which contribute to the genetic variance indexed in the externalizing common factor GWAS, may not predict choice selection behaviors, at least among early adolescents.

PGS reflecting inhibition (i.e., sensation seeking, negative urgency, positive urgency, perseverance, and premeditation) and impulsivity (i.e., attentional impulsivity, motor impulsivity, non‐planning) were also not associated with delay discounting in any of the genetic ancestry groups after multiple testing correction. These findings parallel some studies showing no association between phenotypic delay discounting and indices of impulsivity (e.g., premeditation, sensation seeking) among adults [29]. Results are also in line with work indicating that delay discounting is genetically separable from impulsivity factors with distinct biological mechanisms and small to modest genetic correlations between delay discounting and inhibition and impulsivity metrics [73]. There are several reasons that may explain these null results. The GWAS summary statistics that we used to generate PGS for impulsivity were based on self‐reports. It is possible that self‐report questionnaires of impulsivity tap different processes than those detected via behavioral tasks [74, 75]. While decision making tasks may require participants to make decisions and hypothetical consequences of that decision (i.e., receipt of a reward now vs. later), self‐report measures often inquire about one's affective, cognitive, and behavioral propensities and inclinations on average or over a period of time. Participants' self‐report measures of their predispositions may be biased as their perceptions may not always accurately reflect their behavior. Thus, differences in measurement methods (self‐reports vs. behavioral tasks) may explain why genetic variants associated with self‐reports of impulsivity were not predictive of phenotypic delay discounting, although future research further interrogating these relationships is needed.

It is notable that none of the PGS under study were associated with phenotypic delay discounting among participants most genetically similar to AFR and AMR reference populations after multiple testing correction. The lack of statistically significant PGS‐phenotypic delay discounting associations may be due to an ancestral mismatch between our target sample and the discovery samples, which is known to be critically important for genetic prediction. Numerous empirical studies have shown that cross‐population genetic prediction is often attenuated as the genetic distance between the target population under study and the GWAS discovery sample increases [76, 77, 78]. In addition, the ABCD participants that were genetically similar to AFR and AMR reference panels were much smaller compared to participants that were genetically similar to EUR reference populations; thus, we had less power to detect significant effects.

Finally, we found fewer statistically significant correlations between the PGS and delay discounting outcomes when we applied the less conservative data quality filter (i.e., more than one non‐systematic reversal) and no significant results remained after applying the more conservative filter (i.e., any non‐systematic reversals). Recent findings suggest that rates of non‐systematic discounting data may be associated with myriad individual‐specific and environmental predictors (e.g., cognitive‐task performance; Gelino et al. *in preparation*). It is plausible that the removal of non‐systematic responses via data quality filters resulted in less diversity in key variables across the assessed sample (Gelino et al. *under review*) [79]. Data examining participant characterization based on non‐systematic discounting is limited, and more research is required to better understand how exclusionary measures affect ecological validity and impose bias in choice research.

There are some limitations of the study to acknowledge. As noted above, PGS for delay discounting, educational attainment, externalizing symptoms, inhibition, and impulsivity were based on GWAS that consisted of individuals of European descent. To date, the majority of GWAS include predominantly individuals of European ancestry, which has been recognized as a significant problem that has the potential to exacerbate health disparities and impede the equitable implementation of precision medicine initiatives [76]. Future genetic identification efforts are sorely needed on more ancestrally diverse populations to ensure that genetic discoveries are relevant to all populations. In addition, the GWAS we used to create PGS for delay discounting was derived from adults as there are no GWAS to our knowledge that have been conducted in child or preadolescent populations. Future GWAS identifying genetic variants associated with delay discounting in preadolescents are needed, which may boost the predictive power of delay discounting PGS.

In summary, we found statistically significant, but modest, associations between delay discounting and educational attainment PGS with phenotypic delay discounting metrics among individuals most genetically similar to EUR reference populations. Given the small effect sizes observed, the translational utility of PGS in terms of influencing delay discounting is limited. The small effect sizes observed of the PGS on delay discounting are not entirely surprising, especially at developmental timepoints where a studied trait is highly variable or malleable. Future work should consider examining the impact of social ecological exposures on phenotypic delay discounting, which may better account for variance in this outcome during preadolescence. Although some studies have found that aspects of the environment (e.g., family psychopathology) [80] are not linked to delay discounting among preadolescents, other environmental variables (e.g., economic and social deprivation) [81] have been robustly associated with delay discounting during this developmental period. Future studies that examine the joint contributions of genetics and environmental exposures on phenotypic delay discounting are needed, as they may provide more translational opportunities aimed at targeting delay discounting and promoting positive health outcomes across the lifespan.

## Conflicts of Interest

The authors declare no conflicts of interest.

## Supporting information


Data S1.

## Data Availability

Individuals interested in accessing the ABCD data are required to submit a data use agreement via the NIMH Data Archive.
