# Supplementary material for: Genetic Propensity for Delay Discounting and Educational Attainment in Adults Are Associated With Delay Discounting in Preadolescents: Findings From the Adolescent Brain Cognitive Development Study
Source: Genes Brain Behav. 2025 Mar 27;24(2):e70020. doi: 10.1111/gbb.70020 (PMC11949538; doi:10.1111/gbb.70020)
Supplement: Supplementary file 1 — Data S1. [file GBB-24-e70020-s001.docx]

**Supplementary Materials**

*Description of Monetary Choice Questionnaire Used to Measure Delay Discounting in GWAS conducted by Thorpe et al. (under review)*

Over the course of approximately 4 months in 2015, over 134,000 individuals responded to survey questions as part of a study on the genetics of decision-making developed by A.A.P. and S.S.R. This included a 30-item monetary choice questionnaire (**MCQ**) we previously employed, which was modified from the well-established 27-item MCQ. The original 27 items presented individuals with a choice between a smaller immediate reward and a larger delayed reward at one of three magnitudes (small: $25–35; intermediate: $50–60; large: $75–85). Additionally, 3 items were introduced to the original 27-item MCQ specifically aimed at excluding inappropriate responders. The questions were as follows:

| Item | Would you rather have: | |
| --- | --- | --- |
| 1 | $54 Today | $55 in 117 Days |
| 2 | $55 Today | $75 in 61 Days |
| 3 | $19 Today | $25 in 53 Days |
| 4 | $31 Today | $85 in 7 Days |
| 5 | $14 Today | $25 in 19 Days |
| 6 | $47 Today | $50 in 160 Days |
| 7 | $15 Today | $35 in 13 Days |
| 8* | $55 Today | $85 Today |
| 9 | $25 Today | $60 in 14 Days |
| 10 | $78 Today | $80 in 162 Days |
| 11 | $40 Today | $55 in 62 Days |
| 12 | $11 Today | $30 in 7 Days |
| 13 | $67 Today | $75 in 119 Days |
| 14 | $34 Today | $35 in 186 Days |
| 15 | $27 Today | $50 in 21 Days |
| 16 | $69 Today | $85 in 91 Days |
| 17* | $60 Today | $20 Today |
| 18 | $49 Today | $60 in 89 Days |
| 19 | $80 Today | $85 in 157 Days |
| 20 | $24 Today | $35 in 29 Days |
| 21 | $33 Today | $80 in 14 Days |
| 22 | $28 Today | $30 in 179 Days |
| 23 | $34 Today | $50 in 30 Days |
| 24* | $15 Today | $35 Today |
| 25 | $25 Today | $30 in 80 Days |
| 26 | $41 Today | $75 in 20 Days |
| 27 | $54 Today | $60 in 111 Days |
| 28 | $54 Today | $80 in 30 Days |
| 29 | $22 Today | $25 in 136 Days |
| 30 | $20 Today | $55 in 7 Days |

*These items were not part of the original MCQ. We added them to identify individuals who responded carelessly. Participants choosing one or more lower monetary rewards for items 8, 17 or 24 were presumed to be responding carelessly and excluded from all analysis.
